# The Effect of Specific Energy Density on Microstructure and Corrosion Resistance of CoCrMo Alloy Fabricated by Laser Metal Deposition

**DOI:** 10.3390/ma12081321

**Published:** 2019-04-23

**Authors:** Jinbao Li, Huijiao Ren, Changsheng Liu, Shuo Shang

**Affiliations:** 1 School of Materials Science and Engineering, Northeastern University, Shenyang 110819, China; jinbaoli@stumail.neu.edu.cn (J.L.); huijiaoren@163.com (H.R.); csliu@mail.neu.edu.cn (C.L.); 2 Key Laboratory for Laser Application Technology of Liaoning Province, Northeastern University, Shenyang 110819, China

**Keywords:** laser metal deposition, cobalt-based alloy, morphology, corrosion resistance

## Abstract

With the development of modern medical implants, there are significantly increasing demands for personalized prosthesis. Corrosion-resistance and dense cobalt alloy specimens have been successfully fabricated by laser metal deposition. The relationship between specific energy density, microstructure and corrosion resistance of the specimens is investigated. The results show that higher specific energy density promotes the formation of columnar grain and leads to coarse grain size. The evolution and distribution of deposited microstructure from bottom to top are summarized in a metallographic sketch. The corrosion current of deposited specimens increases from 2.071 × 10^−6^ A/cm^2^ to 6.86 × 10^−5^ A/cm^2^ and rapidly drops to 9.88 × 10^−7^ A/cm^2^ with increase of specific energy density from 318.8 J/g to 2752.3 J/g. The columnar and equiaxed structure of deposited specimens have lower corrosion current than mixed structure due to finer grain and less Mo segregation. The deposited have low level metal released because of passive film. The passive film have different formation routes in Hank’s solution and acidic saliva. The specific energy density has an important effect on the microstructure of deposited, which improves corrosion resistance and life span in implant.

## 1. Introduction

Cobalt-chromium-molybdenum (CoCrMo) alloy is widely used in removable partial dentures and medical implant such as metal frames, customized abutments, crowns, bridges, hips, knees and ankles [1,2]. Although the stiffness of the CoCrMo alloy differs from that of human bone, CoCrMo alloys have particular advantages in medical implant for its excellent wear and corrosion resistance. Currently, around 10% of joint transplant patient total hip arthroplasty require revision surgery [3]. This proportion has been continuously increasing with the younger and more physically active group at a higher revision rate. These revisions are caused by a mismatch between implant and bone geometry and corrosion of implant. Additive manufacturing (AM) is a flexible manufacturing technology, and includes selective laser melting (SLM), electron beam melting (EBM) and laser metal deposition (LMD). The near-net-shape achieved by AM can shorten delivery time for components, thus enabling mass production of CoCrMo alloys part in a cost effective manner. Customized components can be achieved through freedom of CAD design and increasing innovation [4]. Geometric accuracy is also improving as AM techniques continuously develop.

Laser metal deposition (LMD) is an additive manufacturing technique that delivers metal particles to the location of a melt pool formed by a laser beam [5,6]. It consists of a coaxial nozzle and NC machine tool, a cooling water machine and shielding gas to protect the molten pool created by a laser beam. With this layer by layer increment manufacturing process, the repair and production of metallic components, smart structures, and functionally graded materials can be achieved for applications in aerospace, biomedical and automobile industries [7,8,9]. SLM and LMD share similar fundamentals of high energy density heat sources, localized melting and microstructural evolution based upon solidification of the melt. SLM has advantages of fine surface quality and is suitable for complex internal features. However, LMD has higher efficiency and low cost per item due to its higher scanning speed and larger beam spot. It is a promising approach to solve the issues of high manufacturing cost, the mismatch between implant and bone geometry, long product cycle and biocompatibility for CoCrMo implant [10,11].

In recent years, the optimization process parameters have been investigated in order to obtain full dense parts and improve mechanical properties. Do-Sik Shim et al. [12] investigated the relationship between the energy density, the powder feed rate and the single-layer height of deposited tracks with 31 sets of process parameters. A method for selecting an appropriate layer thickness setting is proposed through a feedback control process. Chongliang Zhong et al. [13] recommended several methods to reduce the porosity of the deposited based on the influence of laser power, nominal powder particle size, and on the porosity of laser deposited single tracks in LMD. 

Porous structure and small-scale deposition have attracted attention due to the need for refinement and weight reduction. S. L. Campanelli et al. [14] designed a different relative density lattice structure of Ti6Al4V fabricated by SLM. The result shows that a higher energy density of laser beams causes the formation of martensitic alpha leading to higher micro-hardness. The statistical optimization was used to identify the optimal geometric configuration that maximizes peak strength and energy absorbed per unit mass. Jesús del Val et al. [15] have deposited a thin and narrow fine line with 14 μm wide and 7.2 μm thick by side powder injection laser micro-cladding technique. Laser irradiation and processing speed have great influence on the main geometrical features of the track. A processing map has been established to point out the working window for the process.

However, the solidification condition of LMD is different from those of traditional process due to its rapid melting and cooling rates (10^6^–10^7^ °C/s). Fine micro-structure, non-equilibrium phase, and anisotropy of mechanical properties have been investigated according to the influence of process parameters on melt pool that is considered as a common phenomenon in metal additive manufacturing process [16]. The unique solidification condition and reasonable design of porous structure give the deposited samples excellent mechanical properties such as compressive and fatigue properties [17,18]. 

For CoCrMo parts, multiple researches focused on the microstructure and its mechanical properties between AM and casting. Furthermore, the cardiovascular stents using a CoCr alloy was successfully fabricated by SLM which has similar surface roughness and micro-hardness compared to macro SLM components. It is a potential approach that can be treated as an alternative conventional manufacturing cycle based on microtube manufacturing and laser microcutting [19]. CoCrMo compound fabricated by AM technology usually consists of γ (fcc) and ε (hcp) phase with an intricate network of thin ε-lamellae distributed inside the γ phase. It has a higher hardness than the specimen fabricated by other technique, leading to good wear resistance [20]. E. Liverani [21] had designed three scan strategies to achieve fully dense with superior mechanical strength component. The subsequent kinematic tests, carried out on a cadaver leg, confirmed that the custom-fit articular surfaces sufficiently reproduced natural joint motion. Lin Wu et al. [22] examined the mechanical properties of Co-Cr alloy fabricated by SLM and investigated the correlation between its microstructure and mechanical properties. It is found that the SLM alloy had a dense and obviously orientated microstructure, which led to excellent mechanical properties. The mean yield strength 884.37 MPa of the SLM alloy was notably higher than that 758.73 MPa of currently used cast alloy. 

The deposited CoCrMo specimen with excellent mechanical properties is suitable for medical implantation. Therefore, there is an increasing interest in its corrosion. Li Zeng et al. [23] tested corrosion properties with LMD and cast sample. The result shows that both samples have similar corrosion property in test solutions (pH, 5.0 or 2.5). Yolanda S. Hedberg [24] found that SLM CoCrMo alloy has higher corrosion resistance and lower metal release compared to casting, because the non-equilibrium microstructure of cellular structure that enriched in Mo at cell boundaries suppress carbide precipitation and form martensitic (hcp) phase at the surface. Xin et al. [25] investigated surface characteristics and corrosion resistance of selective laser melted Co-Cr alloy before and after porcelain-fused-to-metal firing. The samples display similar corrosion behavior before and after firing in modified Fusayama artificial saliva. SLM specimens displayed significantly better corrosion resistance than cast specimens in modified Fusayama artificial saliva with pH 2.5.

The corrosion resistance of specimen fabricated by additive manufacturing is better than that of casting. However, there is still significant variance in corrosion properties between specimens [26,27]. The variance affects the lifetime and safety usage of implants. Thus, it is important to further investigate the relationship between fabrication parameters, microstructure and corrosion resistance changes of LMD specimens. In this study, the deposited specimens are fabricated to investigate the influence of specific energy density on microstructure and corrosion resistance. The amount of metal released is used to evaluate biocompatibility as well. The aim was to optimize corrosion resistance through process parameters, reveal the formation of passive film in two simulated body fluids and promote LMD industrial applications. 

## 2. Materials and Methods 

### 2.1. Experimental Materials and Equipment

**Figure 1:**
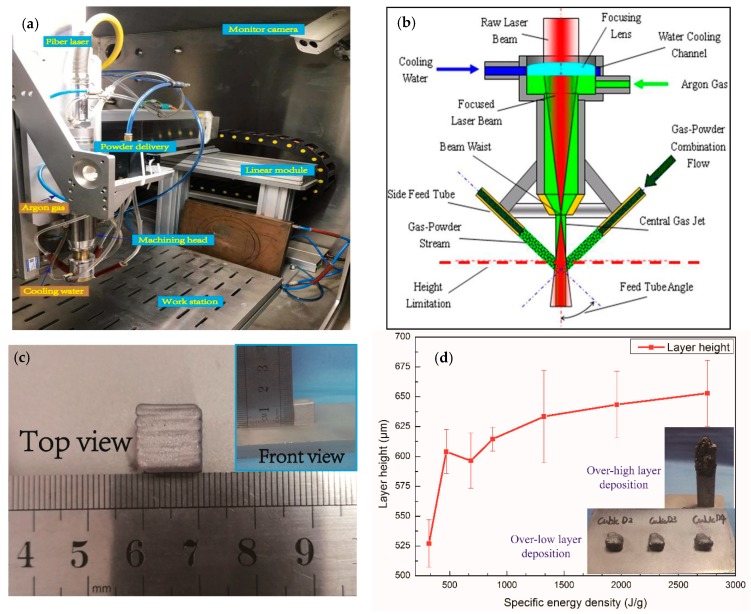
(**a**) The schematic of Laser metal deposition LMD processing assembly; (**b**) Structural sketch of machining head; (**c**) Macro-photograph of the deposited specimens; (**d**) The influence of specific energy density on actual layer height (bottom insert graph: the fail specimen with unreasonable parameters in previous work).

The LMD system (RtPrint1001, Raytech automation Co, Ltd., China) consists of a fiber laser (Rofin fl015c) that serves as the heat source, a numerical control system, a 4-axis NC machine tool and a powder delivery system consisting of one hopper and four coaxial powder nozzles. The beam spot diameter is 1.6 mm that measured a beam quality M2 < 1.3 with Gaussian distribution. The hatch distance of specimen is 1 mm. The machining head, equipped with coaxial powder delivery system, is integrated with an optical system including a collimator and a protective lens to feed the powder coaxially with the laser beam on the substrate surface, placed 9 mm from the nozzle tip. The diameter of feeding fiber core size and divergence value of fiber laser is 100 µm and 33.817 mrad. The laser gets through the collimating (focal length: 80 mm, diameter: 38 mm) and focusing lens (focal length: 125 mm, diameter: 25 mm) to from the molten pool on the substrate. Argon is used as shielding gas from the middle of machining head with 7 L/min as well as carrying gas for the injected powder with 3 L/min. The elevated height of each layer for machining head ΔZ is 0.5 mm. The schematic of LMD processing assembly and a structural sketch of machining head is shown in Figure 1a,b. 

The substrate materials are 316L stainless steel with the dimension of 100 mm × 60 mm × 8 mm. The gas atomized powder produced by Shenyang Research Institute of Nonferrous Metals is used for the deposition. The powder particles size is in the range of 50–150 µm. The composition of powder and substrate are shown in Table 1. The deposited specimens were fabricated on various specific energy with the dimension of 10 mm × 10 mm × 10 mm, as shown in Figure 1c. The various specific energy density is designed to fabricate specimens and process parameters of the experiment are shown in Table 2. The specific energy reflects thermal condition and history of the specimens, as well as the relationship between input energy and feeding rate. Figure 1d shows that the actual layer height is proportional to specific energy density. The actual layer height is kept in an acceptable range from 527.1 μm to 652.9 μm. However, excessive specific energy density results in capturing more particles that gives a broad layer thickness according to our previous work. When the actual layer thickness mismatch with a setting height of machining head (Δz = 0.5 mm), the laser beam and powder flow cannot focus on one point. Thus, the process will fail because over-high actual layer thickness makes the machining head touch the specimen or over-low actual layer thickness makes the specimen no longer grow, as shown in Figure 1d.

### 2.2. Microstructure and Phase Analysis

The specimens are cut, embedded, grinded and polished with diamond paste. The samples are etched with etching solutions (CuSO4:HCl = 4.05 g:20 mL), and it is performed on a field emission scanning electron microscopy (SEM, JSM-6510A, Japan Electronics Co. Ltd.) and optical microscope (OM, GX71, Olympus Co. Ltd.) for microstructural investigation. The SEM equipped with electron probe X-ray microanalysis for analyzing micro–component. In order to identify the phase constitutions, X-ray diffraction (XRD, Japan Electronics Co. Ltd.) was used with Cu Kα generated of 40 kV, 40 mA and a scanning speed of 4 deg/min between 30° and 100°. Group A and B are parallel group and applied with parameters from 1# to 7#, as shown in Table 2. They were used for grain size and grain type statistics. Interception method is selected to measure the average grain size for three random visual fields on each specimen according to GB T 6394-2002 standard. 

### 2.3. Corrosion Resistance Analysis

The specimens are embedded in self-curing epoxy resin, polished with diamond paste and ultrasonically cleaned in ethanol and de-ionized water. Three groups of specimens are immersed in Hank’s and acidic saliva at 35 degrees centigrade for seven days, 15 days and 30 days respectively. All samples are used for each corrosion property test, carried out with an electrochemical workstation (CS350, WuHanKeYi Co. Ltd.). Electrochemical cell uses a three-electrode system, with a saturated calomel electrode (SCE) as the reference electrode, platinum foil (Pt) electrode as the counter electrode and specimens as the working electrode. The electrolyte is either Hank’s simulated body fluid or acidic saliva. The test starts with a potential scan rate of 5 mV/s at room temperature after the prepared sample potential to be stable for 15 min.

Inductively coupled plasma mass spectrometry is used to measure ion content and XPS is utilized to identify the constituents and chemical composition of specimen surfaces with soaking seven days. The XPS spectra are background subtracted with the Shirley method. The analysis is carried out using a monochromatic Al Ka electrode at 1486 eV and 150 W. The acidic saliva and Hank’s solution are prepared in accordance with ISO/TR 10271 [28] standard as shown in Table 3. The volume of solution in metal release test is calculated with EN ISO 10993-12:2004 standard [29]. 

## 3. Results & Discussion

### 3.1. Morphology and Phase Analysis

**Figure 2:**
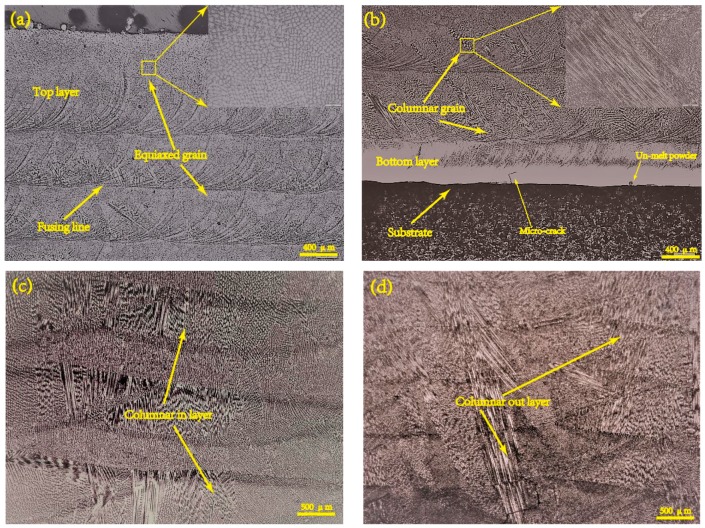
Optical micrograph of deposited specimens in (**a**) top area and (**b**) bottom area; two types of columnar grain (**c**,**d**) in middle area.

Figure 2 shows the solidification morphology of the deposited structure. It indicates that the deposited microstructure consists of cellular grain, columnar grain and equiaxed grain. Columnar grain and equiaxed grain are the predominant microstructure of the deposited specimens, in addition, cellular grain at the bottom. The top part and interlayer junction of specimen’s cross section are equiaxed grain, as depicted in Figure 2a. The top deposited layer is thicker than others because of remelting phenomenon. Columnar grain grows from the bottom of each layer, especially some columnar grain with elongation pattern and vast size, as shown in Figure 2b. The microcrack is also observed on the interface between substrate and the deposited due to the difference of thermal expansion coefficient between substrate and the deposited. The types of columnar grain are distinguished according to whether penetrating the interlayer. They depend on epitaxial growth which is affected by specific energy inputs. Columnar grain disrupted by equiaxed grain each layer in the middle area, like a sandwich structure and the vast size and penetrating the interlayer columnar grain, as shown in Figure 2c,d. The temperature gradient descents and solidification rate increases from bottom to top results in the microstructure transformation from cellular grain to equiaxed grain. If energy input is enough to remelt the equiaxed area of underlying layer with the specific energy density increase, the columnar grain will continuously grow by epitaxial growth. On the contrary, if the specific energy density is too low to remelt, the columnar grain will be blocking up. Furthermore, the increase in solidification rate leads to grain refinement.

**Figure 3:**
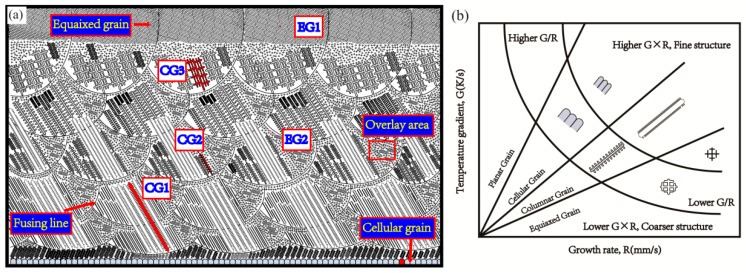
(**a**) The microstructure sketch of deposited specimen; (**b**) Effect of temperature gradient G and growth rate R on the morphology and size of solidification microstructure [32].

Figure 3a illustrates a sketch microstructure map of deposited specimens. Descent in temperature gradient and increase in solidification rate both contribute to the increase of constitutional super-cooling, resulting in diverse dendrite grain morphology. Temperature gradient G and growth rate R have influence on the morphology and size of solidification microstructure, as shown in Figure 3b. As the simulation result of multi-layer deposited [30,31], G/R and G × R is 177 s/mm^2^ and 6010 K/s at first layer where its second dendrite arm spacing is 1.9 μm. The G/R and G × R is 154 s/mm^2^ and 3980 K/s at the sixth layer where its second dendrite arm spacing decreased to 3.1 μm. The developed primary dendrite, smaller second dendrite arm spacing and coarsening second dendrite will occur in a sequential order like CG1, CG2, CG3 areas in which second dendrite arm spacing increases along with deposited process continue. Moreover, the columnar grain orientation roughly is parallel to build direction. It is notable that overlapped areas consist of equiaxed and columnar grain. In this case, the equiaxed grain exhibit cross-shape like EG1, which differ from polygonous equiaxed grain on top area like EG2.

**Figure 4:**
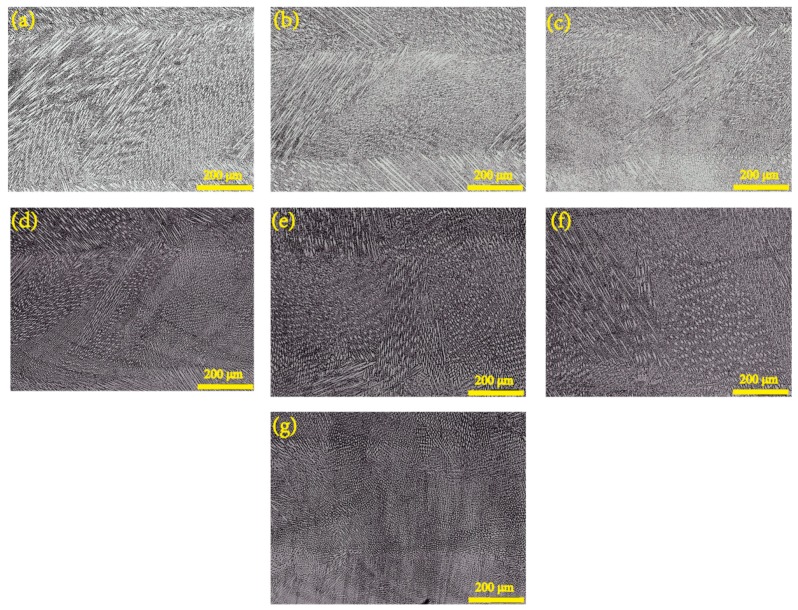
Optical micrograph of deposited specimens on various specific energy density. (**a**) 2752.3 J/g; (**b**) 1960.8 J/g; (**c**) 1323.3 J/g; (**d**) 875.9 J/g; (**e**) 684.9 J/g; (**f**) 470.1 J/g; (**g**) 318.8 J/g.

**Figure 5:**
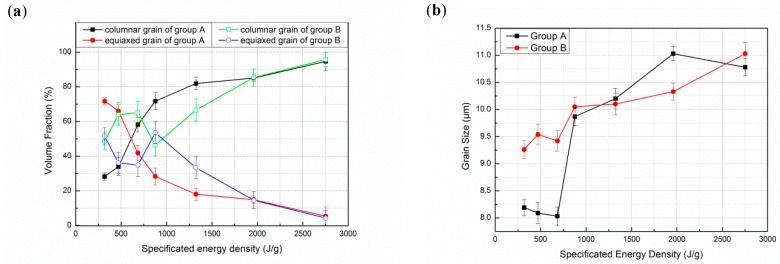
(**a**) The relationship between specific energy density and volume fraction; (**b**) The relationship between specific energy density and grain size.

Figure 4 shows optical micrograph of specimens on various specific energy density from 318.8 J/g to 2752.3 J/g. The microstructure of deposited specimen reflects its local thermal history and solidification process. Through statistics volume fraction of grain, the volume fraction of equiaxed grain decreases as specific energy increases in Figure 5a. The volume fraction of equiaxed grain rapidly descents from 71.73% to 5.35% in the specimen of group A, and the volume fraction in the equiaxed grain on group B drops from 51.46% to 4.3% as well. It is clear that the volume fraction of columnar grain increases as the specific energy increases, which shows a mechanism for competitive growth between columnar and equiaxed grains consistent with published study [33]. Grain morphology and size are influenced by nucleation and growth condition. The condition is strongly related to its thermal history. If laser power is a definite value, the input energy will be constantly used for melting powder, heating substrate and losing in environment. Thus, specific energy density reflects the degree of surplus energy within stable environment. If specific energy is higher, enough input energy not only melts powder, but also heats the substrate or previous layer. Therefore, temperature gradient of specimens increases resulting in epitaxial growth; on the contrary, it will be beneficial to the heterogeneous nucleation mechanism of equiaxed grain. The microstructure is considered as pure columnar grain if equiaxed grain is lower than 0.99%. Otherwise, it is considered as pure equiaxed grain if it higher than 49% [34]. Combining statistical data of volume fraction, the deposition with specific energy density between 470.1 J/g to 1960.8 J/g can be considered as mixed microstructure, as depicted in Figure 4a–f. Specific energy has a small effect on grain size, as shown in Figure 5b. Higher input of specific energy density results in residual heat accumulation which decreases cooling rate and coarse grain structure is achieved. Grain size of deposited specimens remains between 8.19–10.78 μm. 

**Figure 6:**
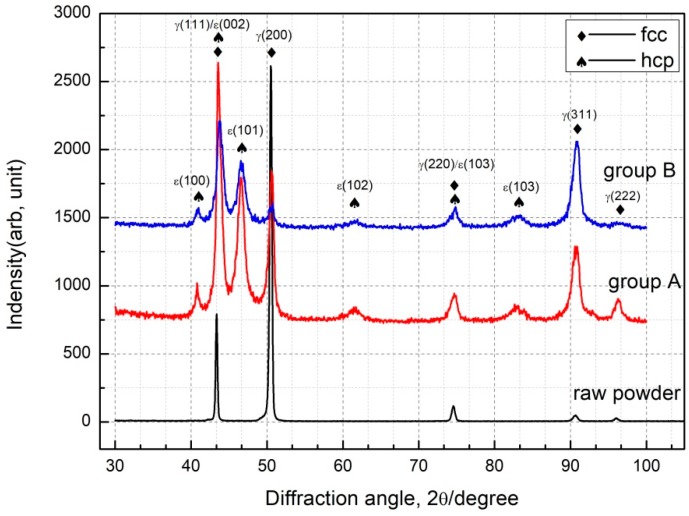
X-ray diffraction spectra of powder and deposited specimen.

**Figure 7:**
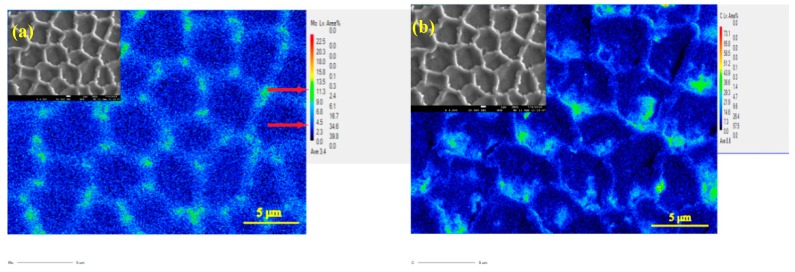
The electron probe micro-analyzer images of (**a**) Mo and (**b**) C.

Figure 6 shows XRD pattern of deposited specimens and powder. The γ-Co phase is observed in all specimens and powder. It is the main phase in deposited specimens and powder, which has an fcc crystallographic structure. However, ε-Co phase peak is also found in the deposited specimens. It is well known that LMD is a layer by layer manufacturing technology. The previous layer will be reheated and approximately cooled to 400 °C as a new layer gets deposited. The ε-Co, hcp structure, is a stable phase that exists from 25 °C to 1250 °C. During deposition, the average temperature of specimens maintains at 400–1100 °C [35]. Therefore, the γ (fcc) phase has enough time to transforms to ε phase during deposition process, usually takes around 30 min. The ε-Co prefers to exist in lamellae shape inside γ-Co, which is consistent with research findings [36]. Due to the constrained dendrite growth under super temperature gradient, the specimen has a strong anisotropy due to its thermal history and inheritance of substrate orientation. Mo mainly distributes on grain boundaries while C mainly adjacent to grain boundary from electron micro probe analysis in Figure 7. The deposited specimens undergo a rapid cooling process resulting in an undercooled melt, which suppresses the formation of micron-sized carbides and the high temperature phase γ will be retained. Thus, the submicron-sized carbides have been found near the grain boundary as shown in Figure 7b, along with segregation of C and Mo at grain boundary in Figure 7. 

### 3.2. Corrosion Resistance of Deposited Specimen

#### 3.2.1. The Effect of Specific Energy Density on Corrosion Current

**Figure 8:**
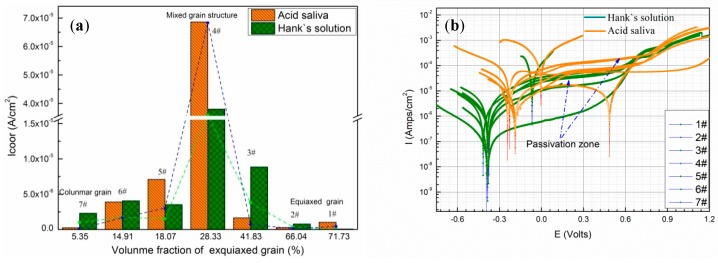
(**a**) Influence of grain structure on corrosion current in acidic saliva and Hank’s solution; (**b**) polarization curves of deposited specimens.

Figure 8a shows the influence of grain structure on corrosion current density in Hank’s solution and acidic saliva. The grain structure of deposited specimens will transform from pure equiaxed grain to pure columnar grain with the increase of specific energy density. The corrosion current density is the highest in both Hank’s solution and acidic saliva with the mixed microstructure, when specific energy density is between 470.1 J/g to 1960.8 J/g. The specimens exhibit passive behavior in both solutions, as shown in Figure 8b. Due to existence of Cl^−^ in Hank’s and acidic saliva, pitting corrosion will occur in passive film. Therefore, current density continually increases after passive area. Higher corrosion current indicates a passive film occur pitting or it is hard to heal. On the one hand, passivation film nucleation depends on the interfacial energy, which promotes passivation film nucleation and growth [37]. The pure equiaxed grain has a finer size with greater grain boundary area than mixed structure that promotes passive film formation and improves resistance to further destruction of film. The corrosion current of a single structure like equiaxed grain is lower, because equiaxed grains promote corrosion resistance [38]. On the other hand, the occurrence of pitting is related to entrainment. Compared with pure columnar grain, mixed structures resulting from lower specific energy density has a lower cooling rate. It is more likely to segregate Mo and from the micron-sized carbides as discussed above. As an important anti-pitting element, Mo segregates between grains and precipitates carbide inclusions, so its corrosion current density increases.

**Figure 9:**
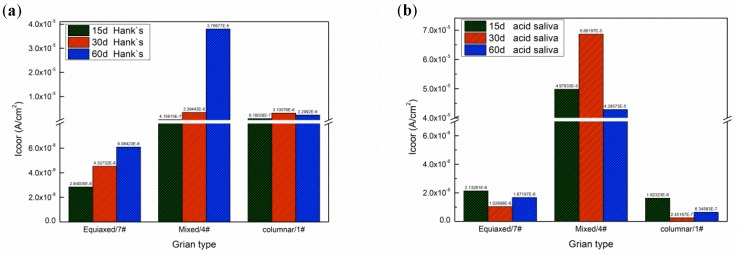
The corrosion current of deposited specimens with different immersion time (**a**) Hank’s solution; (**b**) Acidic saliva.

Figure 8b depicts polarization curves for deposited specimens in Hank’s solution and acidic saliva. The passive region of the deposited specimen in acidic saliva is extended over a wider range of potential, generated dense and more stable passive film than in Hank’s solution. The specimen in Hank’s solution shows a lower corrosion potential. The corrosion current of deposited specimens for different immersion length are shown in Figure 9. The corrosion current density in acidic saliva is generally lower than that in Hank’s solution. As immersion time increases, the current density in the Hank solution gradually rises, while corrosion current density in acidic saliva changes little or even declines. The deposited specimens are more stable in acidic saliva, hence immersion time has little influence on corrosion in agreement with literature study [24]. 

#### 3.2.2. Passive Film Formation Analysis

The amount of released metal for each specific energy density in Hank’s solution and acidic saliva are listed in Table 4. Regardless of the influence of deposition parameters, the released amount of Co is the highest. The amount of Co reaches 1.62 μg/L in acidic saliva, which contains more hydrogen ions, while only reaching 0.604 μg/L in Hank’s solution. This also applied to Mo and Cr. It is notable that deposited specimens on 318.8 J/g, the amount of ion released is much higher than other samples. The average released amount of Co, Mo Cr, Fe is 0.584 μg/L, 0.127 μg/L, 0.006 μg/L and 0.006 μg/L respectively. The amount of Co release level is in the range of 0.214–1.62 μg/L, which is significantly lower than the security threshold suggested by published literature [39,40]. This indicates that deposited specimen is safe in term of ion released.

**Figure 10:**
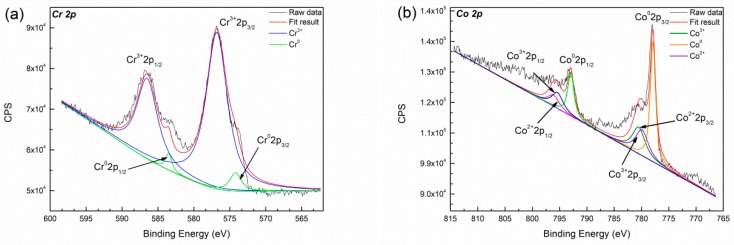

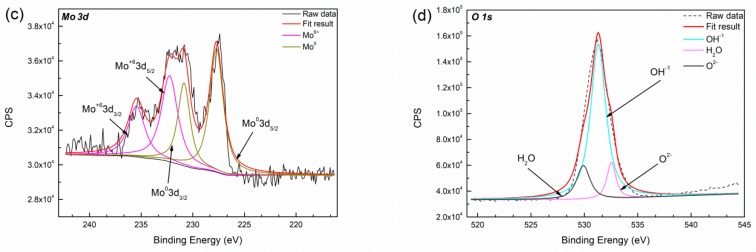
The X-ray photoelectron spectroscopy results of the specimen after immersion in Hank’s solution for seven days (**a**) Cr; (**b**) Co; (**c**) Mo; (**d**) O.

Figure 10 shows the XPS survey spectra of specimen after immersion in Hank’s solution for seven days. XPS spectra of binding energy regions of Co 2p, Cr 2p, Mo 3d, O 1s electrons is obtained from deposited specimens. Each spectrum is decomposed into spectra originating from both metallic and oxide states. The high resolution of binding energy are shown in Figure 10b–d. The peaks spacing between Cr 2p_1/2_ and Cr 2p_3/2_ is 9.7 eV, which revealed the predominate presence of Cr_2_O_3_, while the side of small peak can be identified as Cr^0^. The peaks spacing between Co 2p_1/2_ and Co 2p_3/2_ is 15.8 eV, which is identified as Co, Co^3+^ and Co^2+^ coinciding with published data [41]. The broad asymmetric shape for the O 1s peak indicated the presence of both metal oxides and hydroxides [42]. According to binding energy data [43], the position of the Mo 3d_5/2_ peak in Mo^4+^ and Mo^6+^ oxide should be 229 eV and 232.6 eV, respectively. Therefore, the binding energy obtained is consistent with the formation of Mo^6+^ compound in this study. The surface of deposited specimen contains Co^0^, Co^2+^, Co^3+^, Mo^6+^, Cr^3+^, O^2−^, and OH^−^ with immersion in Hank’s solution. Co and H_2_O from the complex compounds in solution [44], thus the passive films consist of CoO, Co_3_O_4_, CoOOH, Cr_2_O_3_ and minor MoO_3_.

Then, the corrosion process of Co can be modeled as the following:

Co + H_2_O → Co(H_2_O)ads (1)

Reaction 1:

Co(H_2_O)ads → Co(OH)^+^ + H^+^ + 2e^−^ (2)

Co(OH)^+^ + OH^−^ → Co(OH)_2_ (3)

Co(OH)_2_ → CoO + H_2_O (4)

Reaction 2:

Co(OH)_2_ + OH^−^ → CoOOH + H_2_O + e^−^ (5)

3CoO + 2OH^−^ → Co_3_O_4_ + H_2_O + 2e^−^ (6)

**Figure 11:**
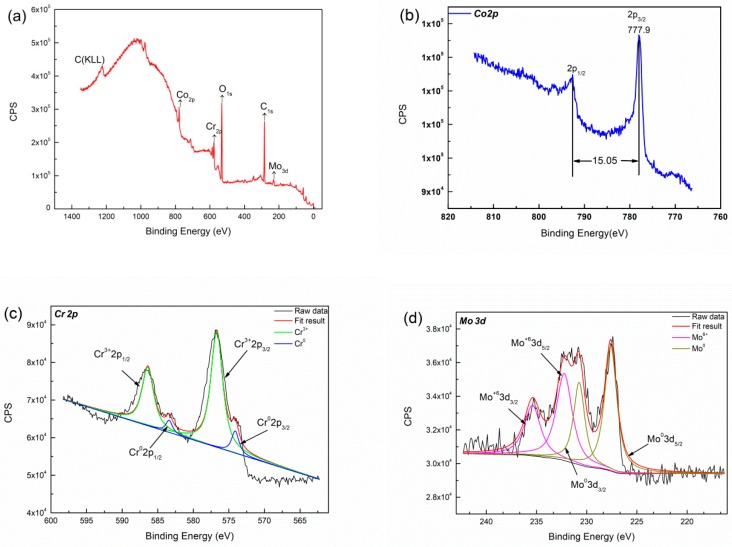
The survey spectra of the specimen after immersion in artificial acidic saliva for 7 days, (**a**) full spectrum scanning; (**b**) Co; (**c**) Cr; (**d**) Mo.

Figure 11b depicts the binding energy of cobalt in high resolution. The peaks spacing between Co 2p_1/2_ and Co 2p_3/2_ is 15.05 eV, which indicates Cobalt does not appear on the surface in the form of ions. The Mo and Cr elements do not show obvious difference with immersion in acidic saliva, as shown in Figure 11c,d. 

Co-based alloy is liable to passivation and shows lower corrosion rates in slightly alkaline or neutral corrosion solutions such as NaOH and NaCl [38]. The specimen also exhibits lower corrosion current in Hank’s solution than those in acidic saliva, as mentioned above. Their results are consistent. However, Co^3+^ is not detected on the surface of specimen after immersed in acidic saliva for seven days. Cr makes a major contribution to passive film formation. The passive films consist of Cr_2_O_3_ and minor MoO_3_ in acidic saliva. The level ion release of Cr in acidic saliva still remains very low 0.002 mg/L, and Co in Hank’s solution is much lower than in acidic saliva. It certifies composition of passive film from another point of view. The stabilized and broadened passivation region is formed in acidic saliva due to the formation of dense, stable and relatively good protective passive film. Therefore, with the increase of immersion time, the corrosion rate of specimen in saliva acid changes little. The passive film contains Co(OH)_2_ will be dissolved in Hank’s solution. 

## 4. Conclusions

This work studied the influence of specific energy density on microstructure and corrosion resistance of Cobalt based alloy specimens fabricated by LMD. The major results are as follows:

(1) The main phases observed in deposited specimens are γ-Co and partially transformed in ε-Co with the deposition processing. Equiaxed and columnar grain have a competitive growth mechanism in deposited specimens. The second dendrite arm spacing of columnar grain increases from bottom to top. Higher specific energy density promotes the formation of columnar grain and leads to coarse grain size.
(2) The corrosion current of deposited specimens increases from 2.071 × 10^−6^ A/cm^2^ to 6.86 × 10^−5^ A/cm^2^ and rapidly drops to 9.88 × 10^−7^ A/cm^2^ with increase of specific energy density from 318.8 J/g to 2752.3 J/g. The columnar and equiaxed structure of deposited specimens have lower corrosion current than mixed structure due to finer grain and less Mo segregation.
(3) The deposited specimens in acidic saliva forms a more stable, dense passive film than that in Hank’s solution. The passive film is composed of Cr_2_O_3_ and displays a wide range of passivation in acidic saliva, while it consists of CoO, Co_3_O_4_, CoOOH, Cr_2_O_3_ and minor MoO_3_ and shows a lower corrosion potential in Hank’s solution. The component fabricated by LMD has low level of released metal in both solutions.

Based on rapid solidification of thermal and material transmission in LMD, the specific energy density reflect the relationship between input energy and feedstock powder that has a strong influence on solidification microstructure. It is an effective way to simplify the number of process parameters to be evaluated and make a deep understanding for LMD process to improve corrosion resistance of the deposited part. If further normalized parameters can be used to the alloys with complex phase structures and porous structure, it will promote the commercial application and upgrade manufacturing services of LMD technology.

## Figures and Tables

**Table 1:** Nominal chemical composites of the Co21 alloy and substrate in wt.%.

Co21	Cr	Mo	C	Fe	W	Ni	Si	S	P	Co
Content (wt.%)	27.26	8.56	0.24	1.07	0.07	3.58	1.14	0.012	0.002	Bal
316L	C	Cr	Ni	Mo	Mn	S	P	Si	N	Fe
Content (wt.%)	0.016	16.33	10.24	2.05	1.11	0.001	0.019	0.52	0.025	Bal.

**Table 2:** The parameters of laser metal deposition.

Order	1#	2#	3#	4#	5#	6#	7#
Power (W)	900	800	700	600	500	400	300
Powder-Feed rate (g/s)	0.327	0.408	0.529	0.685	0.73	0.851	0.941
Scanning speed (mm/s)	7	7	7	7	7	7	7
Line energy density (J/mm)	128.6	114.3	100	85.7	71.4	57.1	42.9
Powder-Feed rate per unit length (g/(mm×s))	0.047	0.058	0.076	0.098	0.104	0.122	0.134
Specific energy * (J/g)	2752.3	1960.8	1323.3	875.9	684.9	470.1	318.8

* Specific energy density: line energy density divided by feeding rate.

**Table 3:** The components of Hank’s solution and acidic saliva.

**Hank’s Solution**	**NaCl**	**KCl**	**CaCl_2_**	**NaHCO_3_**	**KH_2_PO_4_**	**MgSO_4_·7H_2_O**	**MgCl_2_·6H_2_0**	**Glucose**
Content (g/L)	8	0.4	0.14	0.35	0.6	0.06	0.1	0.4
**Acidic Saliva**	**NaCl**	**KCl**	**CaCl_2_·2H_2_O**		**Carbamide**	**NaH_2_PO_4_·2H_2_O**	**Na_2_S·2 H_2_O**	**-**
Content (g/L)	0.4	0.4	0.795		1.0	0.78	0.005	-

**Table 4:** Release amount of metal in Hank’s solution and Artificial acidic saliva after seven days.

**Acidic Saliva**	**Co(μg/L)**	**Cr(μg/L)**	**Fe(μg/L)**	**Mo(μg/L)**
318.8 J/g	1.62	0.003	0.003	0.278
875.9 J/g	0.214	0.002	0.002	0.047
2752.3 J/g	0.441	0.002	0.003	0.067
**Hank’s Solution**	**Co(μg/L)**	**Cr(μg/L)**	**Fe(μg/L)**	**Mo(μg/L)**
318.8 J/g	0.604	0.018	0.005	0.266
875.9 J/g	0.337	0.005	0.011	0.064
2752.3 J/g	0.288	0.007	0.012	0.038
